# Long-distance communication: Looping of human papillomavirus genomes regulates expression of viral oncogenes

**DOI:** 10.1371/journal.pbio.3000062

**Published:** 2018-11-27

**Authors:** Adityarup Chakravorty, Bill Sugden

**Affiliations:** McArdle Laboratory for Cancer Research, University of Wisconsin–Madison, Madison, Wisconsin, United States of America

## Abstract

High-risk human papillomaviruses (HPVs) are a major cause of cancers. HPVs infect epithelial cells, and viral oncogenes disrupt several cellular processes, including cell division, differentiation, and apoptosis. Expression of these oncogenes is relatively low in undifferentiated epithelial cells but increases in differentiating cells by unknown mechanisms. In a new study, Parish and colleagues unveil how two cellular proteins, CCCTC-binding factor (CTCF) and Yin Yang 1 (YY1), mediate looping of the HPV18 genome, which regulates expression of viral oncogenes in both dividing and differentiating epithelial cells.

## Abstract 2

This Primer explores the implications of a new study that connects viral DNA-looping and transcription of human papillomavirus oncogenes at different stages of the viral life cycle.

A subset of human papillomavirus (HPV) types are responsible for approximately 5% of cancers worldwide. For example, these “high-risk” HPVs caused an estimated 640,000 cases of cancer across the globe in 2012 [1]. High-risk HPVs are associated with cervical, vulvar, vaginal, anal, and penile cancers, as well as head and neck cancers. Two high-risk HPVs, HPV16 and HPV18, cause 70% of cervical squamous cell carcinomas and a quarter of oropharyngeal carcinomas [2].

Two viral oncogenes, E6 and E7, are central to the malignancies driven by high-risk HPVs. The E6 and E7 proteins interact with many cellular proteins, including p53 and retinoblastoma (Rb), respectively, and deregulate several cellular processes, such as cell cycle progression, DNA repair, differentiation, and apoptosis [2,3]. Assays using short interfering RNAs (siRNAs) have shown that knocking down E6 or E7 in cervical cancer cells leads to their death through apoptosis [4,5].

## Differences in E6/E7 expression during differentiation

Levels of E6 and E7 vary through the different stages of the viral life cycle. HPVs infect undifferentiated cells in the basal layer of the skin, oral, and genital epithelia [3]. In these actively dividing cells, viral genomes are maintained, not amplified, i.e., they are replicated in a licensed manner along with cellular DNA [6]. Levels of E6 and E7 are relatively low in these undifferentiated cells, presumably to aid the HPV-infected cells with immune evasion [7,8].

The story changes as epithelial cells move away from the basal layer and start to differentiate. These differentiating cells are the sites of viral DNA amplification and where infectious virions are assembled [3]. However, in the absence of HPV infection, differentiating epithelial cells exit the cell cycle and stop dividing [9]. That would be bad news for HPV because, being a small double-stranded DNA virus with an 8-kb genome, it doesn’t encode its own DNA replication machinery. Instead, HPVs rely on cellular replication factors to replicate or amplify their genomes [6].

So HPV coerces differentiating epithelial cells to re-enter the cell cycle. Levels of E6 and E7 increase in these cells. Among other functions, E7 binds Rb and the pocket proteins p107 and p130, disrupting Rb’s association with the E2F transcription factors. That leaves E2F proteins free to activate E2F-responsive genes and push the differentiating cell into S-phase [2,3,9,10, and references therein]. Expression of E7 alone is enough to restart DNA replication in some in vitro models of HPV infection and epithelial cell differentiation, and expression of HPV16 E7 in the basal cells of transgenic mice also exposed to estrogen led to cervical cancer [11,12].

As E7 drives HPV-infected differentiating epithelial cells back into the cell cycle, E6 interacts with several cellular proteins, including p53. Along with a cellular protein called E6-associated protein (E6-AP)—an E3 ubiquitin ligase—E6 leads to the ubiquitination and degradation of p53 [10]. The E6-mediated degradation of p53 inhibits apoptosis and seems to be important for the production of viral capsid proteins as well [13]. The E6 and E7 proteins can also interact with other cellular proteins involved in cell cycle progression and apoptosis [7 and references therein] and may have yet undiscovered roles in differentiating epithelial cells.

The crucial parts played by E6 and E7 in ensuring a conducive environment for HPV genome amplification in infected cells raises a couple of important, related questions: how do levels of E6 and E7 proteins increase as HPV-infected epithelial cells leave the basal layer and differentiate? How are E6/E7 levels kept low in undifferentiated cells?

## Hints of cellular regulators of E6/E7 expression

Some clues have emerged from previous studies. We know that two HPV proteins—E1 and E2—also regulate the expression of E6 and E7 [14]. In most HPV-induced cancers, viral DNAs are integrated into the cellular genome. Integration usually deregulates E6 and E7 expression and is often linked to the disruption of E1 and E2 expression [15]. Luciferase reporter assays have indicated that several cellular proteins may also be part of the E2-mediated regulation of E6/E7 expression [16].

In the context of the whole HPV18 genome, Joanna Parish and colleagues showed in 2015 that mutating a binding site for the cellular protein CCCTC-binding factor (CTCF) within the HPV genome leads to more E6/E7 protein and increased proliferation of the infected cells [17].

CTCF binds to tens of thousands of sites across the human genome [18]. CTCF has many roles, including regulating transcription and mediating changes in chromosomal architecture and organization [18]. The importance of CTCF is highlighted by studies showing that knocking down CTCF in mice or zebrafish embryos is lethal [19].

CTCF also binds to the genome of several DNA viruses, including Epstein-Barr virus (EBV) and Kaposi’s sarcoma-associated herpesvirus, and is thought to influence viral genome organization and gene expression [20]. In EBV, for example, disrupting specific CTCF-binding sites on the viral genome can alter DNA looping and affect which viral genes are expressed in latently infected cells [21].

Another clue as to how E6/E7 expression is regulated in undifferentiated versus differentiating epithelial cells comes from studies from the 1990s. The promoter region upstream of the E6/E7 genes—often referred to as the long control region (LCR)—in HPV16 and HPV18 contains a silencer region bound by the cellular Yin Yang 1 (YY1) protein [22]. It’s likely that binding of YY1 to this silencer region represses downstream gene expression by excluding cellular proteins, like activator protein-1 (AP-1) [23].

Given that both CTCF and YY1 seem to regulate E6/E7 expression in high-risk HPVs, could these cellular proteins be cooperating to keep E6/E7 levels low in undifferentiated epithelial cells? Could their disruption lead to the high levels of E6/E7 seen in differentiating epithelial cells in which HPV DNA is amplified?

## CTCF and YY1 work together to affect E6/E7 expression

The answers to both the questions above are revealed in a new study by Ieisha Pentland, Karen Campos-León, Parish, and colleagues published in this issue of *PLOS Biology* [24]. The study shows that CTCF and YY1 work together to loop the HPV18 genome and inhibit E6/E7 expression in undifferentiated epithelial cells. As these cells start expressing markers of differentiation, YY1 levels decrease, which disrupts the genomic loop and unshackles E6/E7 expression.

The researchers used human foreskin keratinocytes as proxies for undifferentiated, actively proliferating cells of the basal epithelia. They transfected these cells with HPV18 DNA, and in cells that stably maintained HPV18 genomes, they confirmed a link between YY1 and CTCF protein levels and E6/E7 expression. Knocking down YY1 using short hairpin RNAs (shRNAs) led to a 20-fold increase in E6/E7 transcription. Similarly, shRNA-mediated knockdown of CTCF levels increased E6/E7 mRNA levels.

**Fig 1:**
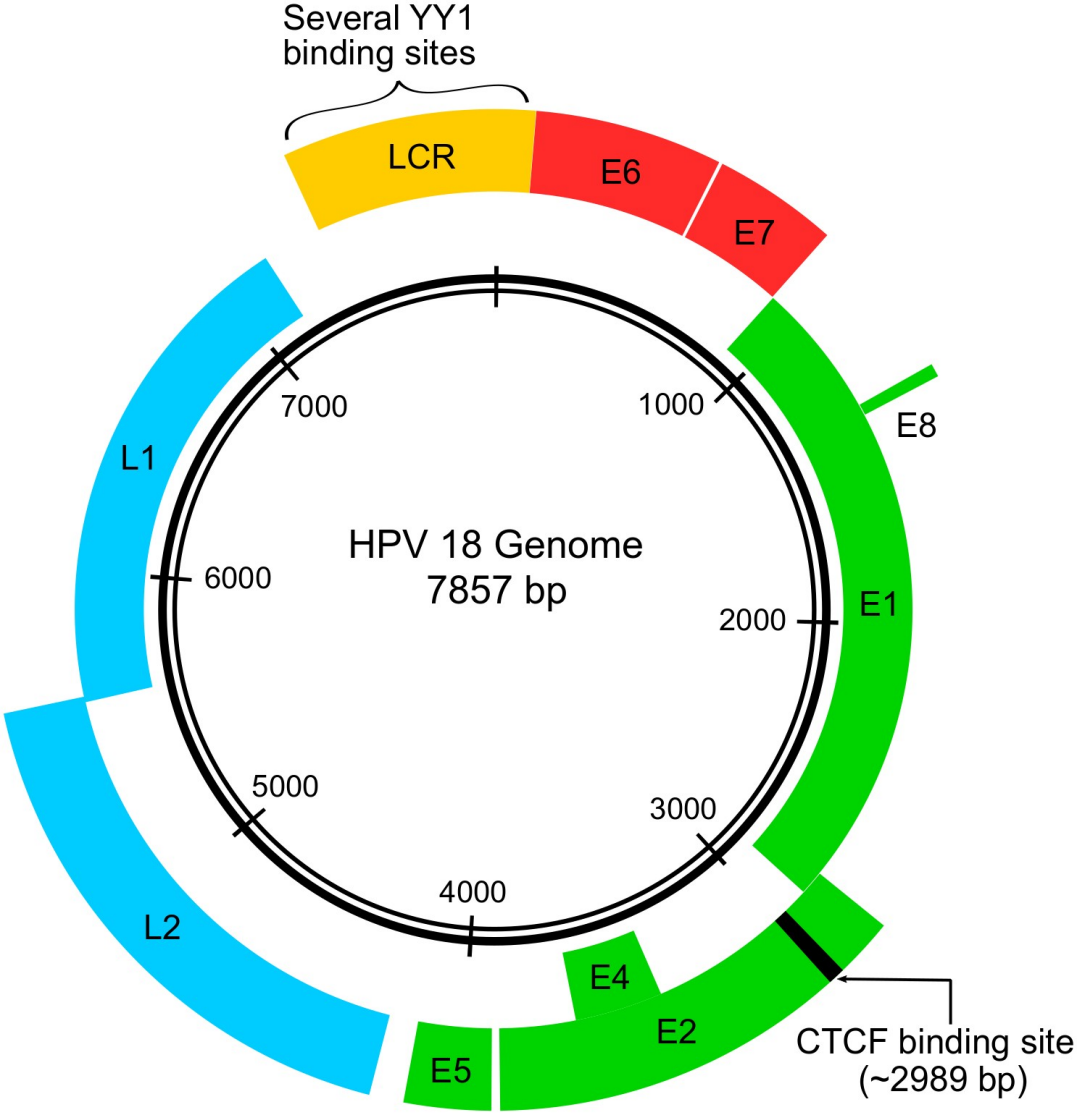
A graphical representation of the HPV18 genome. The E6 and E7 oncogenes are shown in red. The other early genes are shown in green, and the late genes are shown in blue. The LCR, which contains multiple YY1-binding sites, is shown in yellow. The CTCF-binding site in the E2 ORF is also noted (in black). CTCF, CCCTC-binding factor; HPV, human papillomavirus; LCR, long control region; YY1, Yin Yang 1.

More than 3,000 nucleotides separate the YY1-binding sites in the E6/E7 promoter region and the CTCF-binding site in the ORF of the HPV gene E2 (see Fig 1), which is involved in regulating early gene expression and maintenance of viral DNAs. Yet the researchers found that deleting the CTCF-binding site in the HPV18 E2 ORF increased E6/E7 expression in undifferentiated cells (but did not affect levels of E2 itself). Additionally, deleting the CTCF-binding site did not disrupt the maintenance of viral DNAs—HPV genome copy numbers were similar in cells with wild-type HPV18 or with HPV18 in which the CTCF-binding site was deleted (HPV18 ΔCTCF).

How is the 3,000-bp communication gap being bridged to regulate E6/E7 expression in these cells? Chromosome conformation capture (3C) assays showed interactions between the YY1-binding sites in the E6/E7 promoter region and the CTCF-binding site 3,000 nucleotides away. Obviously, this interaction was absent in cells missing the CTCF-binding site, but knockdown assays with shRNAs showed that the both CTCF and YY1 are required for this interaction. In fact, CTCF and YY1 seem to stabilize each other’s binding to the HPV genome. Knocking down either protein led to a) less of the other protein binding to the viral genome and b) fewer interactions between the E6/E7 promoter region and the CTCF-binding site in the E2 ORF.

Thus, the authors linked reductions in levels of YY1 and CTCF to fewer interactions between their binding sites in the E6/E7 promoter region and the E2 ORF, respectively. In turn, they connected fewer interactions between the E6/E7 promoter region and the E2 ORF to reduced expression of E6/E7 in undifferentiated cells. Then, they showed that a similar process appears to be happening in differentiating epithelial cells.

To induce differentiation of the HPV-infected undifferentiated human foreskin keratinocytes, the authors suspended the cells in a semisolid medium. Forty-eight hours after suspension, the cells expressed both a cellular and a viral marker of differentiation. At this time, E6/E7 expression also increased. CTCF levels didn’t change significantly, but YY1 levels decreased. However, binding of both CTCF and YY1 to viral DNAs, as assayed by chromatin immunoprecipitation (ChIP), decreased dramatically in differentiated cells as did the interactions between the E6/E7 promoter region and the CTCF-binding site in E2 ORF, as measured using 3C assays.

## A model and new questions

Based on their findings, the authors proposed a model of how HPV E6/E7 levels are kept low in undifferentiated basal epithelia and increase as epithelial cells move away from the basal layer and differentiate. In basal cells, YY1 levels are relatively high, and the protein binds to sites in the E6/E7 promoter and forms a loop with CTCF bound to a site in the E2 ORF. This DNA loop stabilizes the binding of both proteins to HPV DNA and represses expression of E6 and E7.

When the HPV-infected cells differentiate, YY1 levels decrease and the binding sites in the E6/E7 promoter are vacated. Lower YY1 levels also destabilize CTCF-binding to the viral genome. Fewer loops are formed and E6/E7 transcription increases.

Consistent with this model, the authors used Formaldehyde-Assisted Isolation of Regulatory Elements (FAIRE) to show increased amounts of open/nucleosome-free chromatin around the E6/E7 promoter in both differentiating cells and when YY1 or CTCF protein levels were knocked down in undifferentiated cells. FAIRE involves cross-linking chromatin using formaldehyde, sonicating the crosslinked chromatin, and isolating DNA using phenol–chloroform extraction [25]. The idea is that DNA free from nucleosomes will be purified in the aqueous phase, while DNA cross-linked to histones will segregate to the organic phase.

The researchers also used ChIP assays to show that the repressive epigenetic marker (H3K27Me3) was reduced in the E6/E7 promoter region in differentiating cells and in cells infected with HPV18 ΔCTCF. On the other hand, a marker for transcriptionally active chromatin (H3K4Me3) as well as RNA polymerase II occupancy were increased in both differentiating cells and in cells with HPV18 ΔCTCF compared to wild-type HPV18.

These results highlight the capacity of viruses to influence and co-opt cellular processes for their own benefit. Levels of YY1, for example, are also higher in HPV-negative epithelial cells in the basal layer and decrease as the cells differentiate [26]. In fact, overexpression of YY1 in a human keratinocyte cell line seems to maintain an undifferentiated phenotype in cells even under conditions that would typically lead to differentiation [27]. It remains unclear how the level of YY1 is modulated in undifferentiated versus differentiated epithelial cells. Does HPV influence this process in the cells it infects or is the shift in YY1 expression as epithelial cells differentiate a cellular outcome that HPV has co-opted for its own benefit?

Similarly, several viruses appear to use CTCF-mediated DNA-loop formation during infection. For example, Paul Lieberman’s group used 3C assays to show different DNA loops forming in different latency programs of the Epstein-Barr virus [21]. One such loop between the viral promoter Cp and the viral origin of replication also spanned about 3,000 bp, much like the loops between YY1 and CTCF discovered by Parish and colleagues. In high-risk HPVs, a different CTCF-binding site in the ORF of the late gene L2 was shown to be important for viral genome maintenance and differentiation-induced amplification [28]. Are there yet more roles of CTCF during the HPV life cycle?

As mentioned previously, two viral proteins—E1 and E2—also regulate the expression of E6 and E7 [14]. In most HPV-induced cancers, viral DNAs are integrated into the cellular genome, and the resultant disruption of E1 and E2 expression is thought to deregulate E6 and E7 levels in these cells [15]. However, YY1-binding sites in the E6/E7 promoter region of the HPV16 genome appear to be mutated in several tumors in which the viral DNA persists as episomes [29]. In addition, Parish and colleagues report that a mutation in the CTCF-binding site in the E2 ORF that is predicted to enhance CTCF binding was correlated with lower rates of cancer development. Ultimately, it will be important to determine how the regulation of E6/E7 expression as outlined by Parish and colleagues translates into the context of HPV-driven malignancies.

## Abbreviations

3C: chromosome conformation capture
AP-1: activator protein-1
ChIP: chromatin immunoprecipitation
CTCF: CCCTC-binding factor
E6-AP: E6-associated protein
EBV: Epstein-Barr virus
FAIRE: Formaldehyde-Assisted Isolation of Regulatory Elements
HPV: human papillomavirus
LCR: long control region
Rb: retinoblastoma
shRNA: short hairpin RNA
siRNA: short interfering RNA
YY1: Yin Yang 1
